# Sanatoria in the Light of the Departmental Report

**Published:** 1912-08-24

**Authors:** 


### SANATORIA: THEIR USE AND ABUSE.

**(Contributions, experiences and questions relating to Sanatoria, their Administration and Work, are invited.)**

### Sanatoria in the Light of the Departmental Report.

The interim report of the Departmental Committee on Tuberculosis recently published contains much information of interest to architects who will presently be busy designing sanatoria, which, it is suggested, should be inexpensive and yet attain the high standard required in buildings to combat disease.

### Information Lacking.

Unfortunately, while the interim report deals comprehensively with certain aspects of the question, the information given in respect to accommodation and building generally remains to be treated more extensively in the final report. And probably it was the meagre information available which caused the lamentable *faux pas* made by the Welsh Memorial Committee, as reported in our columns of July 6 (page 341). In this case accommodation for four-fifths of the patients was suggested to be on the open-cubicle system, the partitions not going up to the ceiling, and, though some may favour this, leading authorities favour the open-ward system—the initial cost is less; air circulation, which is so important, is better; and supervision is reduced to a minimum. The Departmental Committee state that, other factors being equal, the larger institutions will have obvious economic advantages; this architects can well appreciate, for the larger the number of beds the lower *pro rata* will be the cost per bed in relation to the administrative blocks, and the planning of an institution must allow for future pavilions. To determine the area of a site—half an acre per patient is suggested as a fair allowance—the site, where practicable, should be somewhat elevated and sloping ground, with a sunny exposure, sheltered from prevailing winds, and with a dry and permeable soil, abundant water supply, and facility for efficient drainage, near a railway station for convenience of transport and haulage, and, if possible, where a low rainfall is registered.

One of the most difficult problems in the whole matter is the necessary segregation of children and the conduct of their education. It is evident the extra accommodation for administrative purposes will increase the apparent cost per bed, which in the selection of competitive schemes is such an important factor. On the other hand, a scheme to be satisfactory must provide all the requirements of an ordinary residential school combined with other special features. It will be interesting to observe how Committees will treat this item; whether distinct institutions or only separate pavilions will be required.

Where new institutions are to be built it would seem that, the site permitting, a central administrative block should have its main axis north and south, flanked on either side by ambulant pavilions for each, six with separate-room blocks on the south aspect to contain the acute cases, averaging about one-fifth of the whole number. These blocks would all be connected by open bridgeways, and the observation or isolation block placed to the north beyond the administrative block. In this connection it is notable that the Departmental Committee state that, provided adequate ventilation is provided, tuberculous patients do not individually require so large a cubic area as other infectious cases, and, further, that treatment of advanced cases does not require hospitals of a special type, it being proposed that accommodation might be provided in hospitals where other diseases are treated.

As one bed per 5,000 of population is suggested for sanatorium purposes, it seems probable that many authorities may elect to set apart beds in existing institutions and thereby save money; but, as the Committee state, the total of new accommodation required is yet problematical, depending on the extent to which existing buildings are or may become available to meet future requirements, the probable number of patients in the different stages of the disease, the degree to which these patients will respond to treatment, and the extent to which Insurance Committees and other bodies may decide to give institutional as distinguished from other forms of treatment. The estimated cost for additional adult sanatoria, including land and administrative quarters, is given at £150 per patient, which seems rather low, and may perhaps be accounted for by the present low value of land in country situations.

